# Hospital and Institutional News

**Published:** 1920-12-11

**Authors:** 


### HOSPITAL AND INSTITUTIONAL NEWS.

### LORD DAWSON ON A STATE SERVICE.

Speaking at the Manchester Town Hall recently, Lord Dawson, who was chairman of the Consultative Council on Medical and Allied Services, pointed out that there is need to determine the policy underlying the present haphazard organisation of medical services. As to whether this should aim at nationalising the doctors, Lord Dawson remarked that the underlying principle of the interim report of the Consultative Council is provision of fabric and equipment, but freedom between doctor and patient. It is felt that nothing should be done to make the family doctor an official, and the patient not a friend but a number. The first to suffer from such a State service will be the workman and his family. Commenting on the lack of support given to hospitals, he proposed that hospitals which have teaching schools shall be put into a separate schedule, and receive some financial assistance from the national exchequer. The Manchester Royal Infirmary he instanced as a hospital which receives patients far and wide because of its eminent teaching school.

### VENEREAL DISEASE.

During the week has been issued the report of the Select Committee of the House of Lords and the House of Commons which was appointed to consider the three Criminal Law Amendment Bills. Taking the Government Bill as a basis, the object has been to evolve for its provisions a single measure securing such alterations in the law as seemed both desirable and necessary. Apparently the case has been strongly put forward that the questions of communication and prevention of venereal disease should be dealt with by a measure directly protecting public health and not under the Criminal Law. We see it stated that according to the Ministry of Health the matter of invoking the aid of Criminal Law in this country is really a question for the Home Secretary, and that the provisions of the Government Bill might have a certain moral and deterrent effect, and, therefore, the Committee have determined to retain Clause 5, with the idea that no difficulty will in practice arise. On the other hand, the question of Clause 7—*i.e.*, that prohibiting advertisement of preventive means—has occasioned such difference of opinion that they have thought it best to reserve any final recommendation and to refer the matter to Parliament. The situation as it stands for the moment is, in view of the Health Ministry's supposed attitude, a little difficult to analyse, but we hesitate to think that the suggestion is to invoke the aid of penal measures against its victims, in place, or partly in place, of a whole-hearted campaign of true scientific prevention of venereal disease.

### INCREASED COST OF TICKETS IN BIRMINGHAM.

The Governors of the General Hospital, Birmingham, met on Friday last to receive a recommendation from the Board of Management that the cost of subscribers' tickets be doubled. This is the first time the price has been revised since 1914, and in moving a resolution that the present price of £2 2s. be increased to £4 4s. the chairman (Mr. C. H. Saunders) drew attention to the fact that the old figure, which was fixed many years ago, was now hopelessly inadequate to meet the cost of treating the patients. The cost of in-patients per week to-day is £4 8s. 5d., and, as the average stay in hospital is approximately three weeks, the revised cost of a ticket represents roughly only one-third of the actual cost of treatment. The chairman explained that annual subscribers of two guineas would be Governors, as heretofore. The resolution was carried unanimously, and the rules of the hospital were altered accordingly. We have never favoured the ticket system because of the hardship it imposes on patients seeking admission; and if it also bears hardly on the hospital, another argument against it has been found. But, surely, if the subscribers value the party patronage which a ticket confers, they should be prepared to pay a fair sum to obtain it.

### MASONIC SUPPORT FOR HOSPITAL.

Most of the Masonic Lodges situated in that part of the Province of Suffolk covering the area of the East Suffolk and Ipswich Hospital have decided to support that worthy institution by annual subscriptions or donations. An emergency Provincial Grand Lodge was held at Ipswich on Monday last to bid farewell to the Provincial Grand Master, the Right Hon. the Earl of Stradbroke, C.V.O., who is leaving to take up his appointment as Governor of Victoria, Australia. The Earl is one of the governors of the East Suffolk and Ipswich Hospital, and during the war he converted his country residence, Henham Hall, into a war hospital, of which Lady Stradbroke was the commandant. A collection was taken for the East Suffolk Hospital, and his lordship promised that the question of a grant should be considered at the next regular meeting of Provincial Grand Lodge. Mr. Arthur Griffiths, the secretary of the hospital, who is well known locally as a Mason, has pointed out to the various Lodges the altered conditions which now obtain in the hospital world—that many who before the war would have gone into nursing homes now perforce have recourse to a general hospital. In short, that the middle classes are now in many instances the real poor, and that this class includes many members of the Craft. If the success which has attended the efforts of this hospital in other spheres of collection is achieved in this direction the result should be a satisfactory accretion to the hospital's income from this comparatively new source.

### FURTHER CO-ORDINATION AT CARDIFF.

We are glad to see that the Executive Committee of the Prince of Wales's Hospital, Cardiff, has welcomed the proposal for closer co-operation between this institution and King Edward VII. Hospital. The Committee declared that in its opinion a medical advisory committee should be found to link all the hospitals which will be supplying clinical material for teaching. This, it held, was desirable in the interest of the Welsh National Medical School. It suggested that the Medical Advisory Committee might consist of the Dean of the Faculty of the Welsh National Medical School, the Professor of Tuberculosis, the Professor of Preventive Medicine, the Chairman of the medical board of King Edward VII. Hospital, the superintendents of the Mental Hospital, Whitchurch, and of Cardiff Sanatorium, the Medical Superintendent of the Hamadryad Hospital, and the Chairman of the honorary surgeons, Prince of Wales's Hospital. Mr. A. Brown and Mr. James Miles, who originated the proposal, from the point of view of King Edward VII. Hospital, must be gratified that it is beginning to bear fruit. When the Prince of Wales's Hospital has finished its work for military patients we may hope that the co-operation will be vigorous, and that thereby the waiting-list at King Edward VII. Hospital will be reduced.

### A BUSY DAY AT CARDIFF.

Mrs. Lloyd George spent a busy day at Cardiff in the interests of the Prince of Wales Hospital for limbless soldiers, of the council of which she is President. She arrived in the Welsh metropolis in the morning, and immediately proceeded to the hospital in The Walk, where she first spent some time in an inspection of various new departments of the institution. Later she was entertained at lunch, when she proposed the Royal toast, that of her own health being given by Mr. Percy Miles, one of the hospital's greatest supporters. Lord Pontypridd added a few words in appreciation of the great work which Mrs. Lloyd George finds time to do up and down the country. In her response, Mrs. Lloyd George announced amid cheers that Mr. Henry J. Lewis, High Sheriff of Glamorgan, and Sir W. R. Smith, Bart., both of whom were present, had each given £1,000 to the hospital, and the Dowager Viscountess Rhondda, who sat near the speaker, £100. This is the hospital's second donation from Mr. Lewis. Afterwards Mrs. Lloyd George performed the opening ceremony of the new departments, including the operating theatre, sterilising room, recreation room, office, stores, and bootmakers' and fitters' shops. In the afternoon the Premier's wife was present at a male voice choir competition in aid of the hospital held at the Cardiff Empire, and presented her husband's gold cup to the choir to which, out of the fifteen contestants, it was awarded by the adjudicator, Dr. Walford Davies.

### COLOURED MILK.

Londoners like their milk coloured, having the delusion that it is of good quality if of a cream yellow. The milk trade is ready to humour this fanciful idiosyncrasy, despite the fact that in December 1917 a "Dora" regulation prohibited the adding of colouring matter to milk. This matter is referred to by the Southwark public analyst in explanation of the higher percentages of milk adulteration, which are really due to the rule that coloured milk is classified as adulterated. As he says, colour is often added to milk of very high quality, and, although this is strictly a form of adulteration, it cannot be looked upon in the same way as the usual methods of adulteration, since it does not lower the quality of the milk. There would be little hope of taking successful legal action for the addition of colouring matter, and in the circumstances he proposes to discontinue classifying coloured milk as adulterated.

### THE TUBERCULOSIS SOCIETY.

As we go to press the annual meeting of this Society is being held (December 10) at the Cabin Restaurant, Tothill Street, Westminster, at 7.45 p.m. Among the agenda we notice a discussion on the election of honorary vice-presidents, the names proposed comprising Sir Clifford Allbutt, and also those of two pathologists. The first will of course be passed *nem. con.*, but it may be that the claims of pathology to still greater honour by tuberculosis workers may strike some as dubious. The last twenty years have witnessed many false hopes raised by pathological tuberculosis work. More than that, the position of this young specialty will not be improved by exalting as authorities those who must necessarily lack a close acquaintance with the disease—in fact, with any disease. The literature of a great subject like tuberculosis is now so vast that no worker in a general laboratory can possibly keep pace with it. And since his clinical equipment is necessarily elementary, his qualifications for initiating research are insufficient. In this matter, the phthisiologist—or any specialist—should dictate to the pathologist, and not the other way about. It is a very juvenile conception of science that makes the connotation include primarily the apparatus of the physical laboratory.

### THE EXPENSE OF THE SICK.

Sundry of our lay contemporaries, impressed by the glaring need of economy somewhere, are endeavouring to meet it at the expense of anti-sickness schemes. Just recently the anti-tuberculosis campaign has come in for criticism (as in *The Times* of November 20 last) as being grandiose and over-costly. The most effective answer to such attacks does not lie within our province, being of course political. Why, however, it may be said, are those Ministries presided over respectively by Dr. Addison and Mr. Fisher the only ones to be assailed? For it is notorious that there are others whose exactions are always cheerfully met, although they spend millions where health and education ask but for thousands. It is quite certain that public opinion is behind the latter in their demands, if not behind the former. The return in the physical well-being warrants all expenditure contemplated to date, and to oppose this constitutes a particularly sinister form of reaction.

### A MODEL OF PROSPERITY.

We have to correct an error in our issue of November 27 (page 189), when we inadvertently alluded to Mr. W. H. Jones in such a way that it would be gathered that he is the secretary of the Longton Cottage Hospital. Of course, Mr. Wm. Atkins is, and has been for twenty-three years, the secretary of this well-managed institution, and Mr. Jones, the secretary of the local Hospital Saturday Fund, while having done good service in an auxiliary capacity for the last four years, has nothing to do with the management of the hospital. The credit for the hospital being in its present enviably solvent and efficient condition is chiefly due to the capable Board of Directors, the honorary medical staff, and the matron, secretary and general staff.

### FRAUD IN A HOSPITAL'S FUND.

The secretary of a branch of a trades union has been sentenced to nine months' imprisonment with hard labour on pleading guilty to stealing a sum of £130, entrusted to him by members of his union for transference to the Council of the North Ormesby Hospital, Middlesbrough. The prisoner had used the money to extricate himself from difficulties arising from betting. His counsel said that he hoped in future to make some restitution, but the judge stigmatised the offence as a despicable form of fraud.

### A "GRAVE" AFFAIR.

The chaplain of St. Bartholomew's Hospital Convalescent Home, Swanley, from 1905 to 1910 was the Rev. C. E. L. Wright, who died on July 6 last at Folkestone. After providing that the fullest medical precautions shall be taken to be sure that he was really dead, Mr. Wright left £500 to Charing Cross Hospital, they to keep in repair and good condition the tomb of himself and wife at Bexley, and £500 to the Royal Albert Edward Infirmary, Wigan, with a similar trust as to the tomb of his father and mother. As, apparently, the legacies are not duty free, the hospitals will receive £450 each, which, invested in some trustee stocks, will produce as much as £27 a year for each hospital. As no man can foresee the future of labour, and what in a few years will be the rates of pay to members of the Grave Gardeners' and Monumental Masons' Amalgamated Societies, these two hospitals may find that they have inherited something in the nature of a worry, and probably will have to consider whether they can safely accept the legacies. For the benefit of our Masonic readers and other students of the occult, it may be recorded that Mr. Wright left his Cardinal's robe, biretta, lace rochet, etc., to the Supreme Magnus and Secretary-General of the Rosicrucian Society in England, for the use of the Chaplain-General of the Society.

### MUNICIPAL AID FOR COTTAGE HOSPITAL.

The Essex County medical officer of health has recently had an interview with the medical superintendent of the Halstead Cottage Hospital regarding the use of the institution. It appears that under the will of the late Mr. Courtauld, there are moneys available for extensions in the direction of an out-patient department and a maternity block, but hitherto the hospital committee has done nothing because of the maintenance cost. Being of opinion that if such extensions were made they could be used by the County Council, the Health Committee of the County Council suggests that a sum not exceeding £100 a year should be paid if the cottage hospital authorities are willing for the proposed extensions to be used as a school clinic and child-welfare centre and dental, ophthalmic, tuberculosis and venereal disease clinic and for the treatment of complicated maternity cases.

### AN EMPIRE HOSPITAL FUND.

Any plan which is calculated to arouse interest and public sympathy with hospital work is worthy of consideration, and there is therefore much to be said in favour of a scheme recently made public which has been suggested by a Mr. W. S. Royston, of Warrington, who thinks that an appeal might be made to every mother in the British Empire to ask each of their children to contribute annually one coin in aid of hospital work, such contributions to be made on one special Sunday in the year, and to form a "British Mothers' Sunday Fund."

### A HOME FOR MENTALLY AFFLICTED OFFICERS.

A good deal of discussion has taken place over the proper quarters for ex-Service men suffering from nervous disorders. Public sentiment has been much against asylum treatment, but some of those most familiar with these institutions have, however, urged that it is only the name which is unpopular, and that no better treatment can be had than the modern asylums provide. A good deal of sympathy will be felt for Sir Frederick Milner's argument in favour of a home for mentally afflicted officers, on whose behalf the Ex-Services' Association enlisted his support. Dr. Hugh Morris, who followed at the recent meeting in aid of funds for the foundation of a home, declared that it was unthinkable that these men should be consigned to an asylum until a preliminary attempt had been made to restore their mental equilibrium. We agree. The asylum is often the inevitable final resort; but a home is needed first, so that recoverable cases may be spared the distress which, rightly or wrongly, attaches in the public mind to experience of the ordinary asylum.

### MADAME PATTI'S DARK HOME.

Opinion is divided concerning the Welsh National Memorial Association's reported purchase of Craig-y-Nos Castle, the romantically placed home of the late Madame A. Patti, the great singer, for use as a sanatorium for consumptives. The price at which it was offered to the Association—namely, £20,000 for the castle and its seventy-five surrounding acres of land—made it a rare bargain, but the contention is that Craig-y-Nos, though situated high among the mountains at the upper end of the Swansea Valley and amid the most bracing atmosphere, is, as its name (the Castle of the Night) implies, one of the darkest places imaginable; very little sunlight ever is seen there. That is a serious drawback to its usefulness as a sanatorium, but the medical advisers of the Association urged that the lack of sunlight should not be allowed to stand in the way of the acquirement of immediately available accommodation which could not otherwise be provided for less than something like £120,000.

### HIGH COST DELAYS BUILDING.

It was stated at the interim court of Governors of the Brompton Hospital for Consumption that a promise of £7,000 from King Edward's Hospital Fund had been made towards the provision of 100 additional beds at the sanatorium for ex-Service men. In view, however, of the high cost of building, the existing uncertainty, and the difficulty experienced in raising anything like sufficient money for the purpose, the Committee have decided with regret to postpone the work until a more favourable time. The practice of requiring a contribution towards the cost of treatment in suitable cases from the patients, their employers and others responsible, continues to give satisfactory results, and a considerable revenue is derived from this source.

### SOME CURIOUS HEALTH INSURANCE POINTS.

A suggestion having been made that a way of augmenting the income of the clergy would be for poor parsons to qualify as panel doctors and so earn a double wage by treating the body as well as the soul, a contemporary refers to the possibility of poor panel doctors, resenting the competition, retaliating by qualifying as parsons. But, after vested interests have been successfully dealt with, the result would surely be the same under each plan. Another curious point arose when a panel doctor recently contended that, as his residence is now more than two miles from his surgery, he is under no obligation to visit any person attached to his panel list in respect of that surgery. The London Insurance Committee failed to see eye to eye with the practitioner, and ordered him to rearrange his hours of attendance.

### DEATHS UNDER AN ANÆSTHETIC IN HOSPITALS.

The Minister of Health has addressed a circular letter to all hospital and infirmary authorities stating that he has had under consideration the report issued in 1910 of the Departmental Committee of Coroners, particularly in reference to recommendation 8, which is as follows: "In the event of a death under an anæsthetic in a hospital or other similar institution there should be a scientific investigation into the actual cause of death conducted by the authorities in the institution." Valuable results would, in Dr. Addison's view, be secured by the co-operation of hospitals and boards of guardians and their medical staffs in action under the cited recommendations, who are already required by the regulations to report such a death to the Ministry, might materially enhance the value thereof by sending with such reports copies of the findings such as are now proposed. Further stating that the records, which should include such particulars as the name and age of the patient, the nature of the disease, and the operation performed, the anæsthetic employed, the name of the person administering it, the period at which death occurred, the result of the *post-mortem* examination, and any other relevant points, will be made available to the Medical Research Council through the Minister.

### FESTINIOG MEMORIAL HOSPITAL.

The Heroes' Memorial Committee has approved a scheme for building a Heroes' Memorial Hospital at Blaenau Festiniog, at an estimated cost of £10,000. Half of this sum has been conditionally promised by the British Red Cross, and the remainder will be raised by private subscription. A site has been acquired facing the vale of Cwmbowydd for £150, and the plans have been drawn, without fee, by Mr. Clough Williams Ellis, M.C., of London. Certain quarrymen have volunteered to clear the site and to quarry the stone for the building. The hospital, which will possess fourteen beds, with power of extension to twenty, will serve twelve neighbouring parishes. Memorial tablets will be placed in the hall, and a small monument in front of the building.

### A VETERAN PUBLIC OFFICER.

Dr. William Gibson, who now retires from various municipal and other appointments at Campbeltown, in Argyllshire, at the age of ninety-seven, is believed to have been the oldest medical practitioner holding a public office. Dr. Gibson took his M.D. in 1854 and his L.R.C.S. (Edin.) the same year, and entered upon public service some twelve months afterwards.

### THIS WEEK'S DRUG MARKET.

A renewal of activities in demand at an early date seems to be very improbable, and many holders of drugs, who had been waiting for a turn of the tide, seem to have come to the conclusion that prices are not likely to recover within the near future. In the face of accumulating stocks it is difficult to see how the downward tendency is likely to be checked so long as buyers hold off. English refiners of camphor have reduced their quotations following on the reduction in the value of crude. Prices of emetin, hematropin, hyoscyamin, eserin, aloin, and chrysarobin are lower. The value of olive oil is maintained. Cod-liver oil has fallen in price, and it is not improbable that it will fall lower. Among synthetic drugs amidopyrin, aspirin, barbitone, benzoic acid, sodium benzoate, phenazone, resorcin, salol, salicylic acid, and sodium salicylate still have a downward price tendency. Ergot of rye is offered at lower rates. The demand for honey is quiet, and values have an easier tendency. Citric acid and cream of tartar are very quiet, and lower in price.

